# Electrochemical and Mechanical Properties of Cathodically Protected X80 Steel in Different Temperature Soil

**DOI:** 10.3390/ma15165526

**Published:** 2022-08-11

**Authors:** Wenhui Liu, Yanbing Meng, Jun Zhao, Wen Wen, Ming Gong, Shixiong Wu, Songmei Li, Mei Yu, Jianhua Liu

**Affiliations:** 1 School of Material Science and Engineering, Beihang University, Beijing 100191, China; 2 PipeChina Science and Technology Institute, Langfang 065000, China; 3 PipeChina North Pipeline Company, Langfang 065000, China; 4 School of Material Science and Technology, Tianjin University, Tianjin 300072, China

**Keywords:** X80 steel, cathodic protection criteria, hydrogen embrittlement, soil corrosion, SSRT

## Abstract

For the application of X80 pipelines in Northeast China, it is important to establish the correct cathodic protection (CP) potential. To achieve this, potentiodynamic polarization; electrochemical impedance spectroscopy (EIS); a slow strain rate test (SSRT); and a scanning electron microscopy (SEM) fracture morphology analysis were carried out for an X80 steel gas pipeline at several temperatures in Heilongjiang Province, China. The results show that the hydrogen evolution potential of X80 steel in soil at different temperatures was about −900 mV (vs. CSE). The generated hydrogen atoms can be adsorbed on the surface of the pipelines to reduce the surface energy, or they can be diffused into the substrate and accumulate to the critical concentration, inducing the decohesion between different structures and generating additional plastic deformation through dislocation motion. With the peak impedance potential as the minimum potential and the hydrogen embrittlement potential as the maximum potential, the CP potential of X80 steel in the soil at 30 °C, 45 °C, and 60 °C ranged from −900 mV to −1100 mV (vs. CSE), temperatures at which the X80 steel does not corrode or cause hydrogen embrittlement.

## 1. Introduction

X80 pipeline steel has been widely used in gas transmission pipelines in China since 2008 due to its excellent welding performance, corrosion resistance, high strength, and good fracture toughness at low temperatures [1,2,3]. Corrosion and stress corrosion cracking are two major degradation mechanisms for buried X80 pipelines. Cathodic protection (CP) combined with coating has been recognized as the most effective and economic mitigation method for the protection of buried pipelines against corrosion [4,5]. However, it is known that the susceptibility of high-strength steels to hydrogen embrittlement (HE) increases with increasing steel grade, and high negative CP potentials enhance the risk of hydrogen embrittlement [6,7,8,9,10,11,12,13,14]. The potential range adopted in service is often the most critical parameter for pipeline cathodic protection. Corrosion tends to occur under inadequate protection conditions; on the contrary, coating peeling or hydrogen-induced cracking can occur in overprotection conditions [15]. 

There have been a number of experimental studies on cathodic protection and the possibility of hydrogen embrittlement for X80 steels under different surrounding media and temperature conditions. The results of an electrochemical experiment indicate that the potential of hydrogen evolution and the optimum CP potential for X80 steel were −960 mV and −800 mV vs. SCE, respectively, in a simulated DaGang soil solution [16]. However, in a simulated Manchurian soil solution, the experimental results show that −850 mV vs. CSE was the optimal CP potential for X80 steel, and HIC occurred under an applied potential of −930 mV vs. CSE [17]. The critical CP range of X80 steel in different soil extracts at ambient temperature was determined to be −800 mV~−850 mV vs. SCE [18]. The slow strain rate test (SSRT) results show that, under a cathodic potential of −1100 mV vs. SCE, there was a potential possibility of hydrogen embrittlement in the simulated soil conditions [19]. The ductility reduction index was 0.65 at 100 mV of cathodic overprotection for X80 steels, indicating a risk of possible hydrogen embrittlement in NS4 solutions [20]. Electrochemical tests and SSRT tests showed that the X80 pipeline steel was in a cathodic protection state at −850 mV. However, at −1000 mV and −1200 mV, strong cathodic polarization promoted a hydrogen evolution reaction. The strong cathodic polarization potential and SRB increased the SCC susceptibility of the pipeline steel and the hydrogen embrittlement (HE) risk [21]. The effects of the cathodic current density on hydrogen embrittlement were also investigated and discussed by some researchers [22]. The results show that increasing the temperature of the solution from 20 °C to 60 °C led to an increased corrosion rate and efficiency reduction in Fumaria officinalis for API X80 pipeline steel [23]. Okonkwo et al. [24] investigated the effect of temperature on the sour corrosion behavior of API X80 steel pipeline and reported that, as the temperature increased from 20 to 60 °C, the sour corrosion resistance of this steel decreased sharply. In summary, despite extensive research, there is still no agreement on the critical cathodic protection criteria for X80 steel at different steel/soil interfaces and temperatures. According to ISO 15589-1 [25], the critical potential limit needs to be documented or experimentally determined.

In this paper, the hydrogen evolution behavior of X80 steel under different CP potentials and at different temperatures in a typical Heilongjiang soil environment was investigated using potentiodynamic polarization and electrochemical impedance spectroscopy (EIS) measurements. SSRT and fracture morphology analysis were also carried out to evaluate the hydrogen embrittlement susceptibility and effect on the mechanical properties. Finally, the cathodic protection criteria are proposed for X80 steel in the Heilongjiang soil environment based on the experimental results.

## 2. Experimental Setup

### 2.1. Sample Preparation

The material investigated in this study was a high-strength pipeline steel X80. The chemical composition of the steel was as follows (wt%): C—0.07; Mn—1.82; S—0.19; P—0.007; S—0.023; Cr—0.026; Ni—0.17; Cu—0.020; Al—0.028; Mo—0.23; Ti—0.012; Nb—0.056; V—0.002; N—0.004; B—0.0001; and Fe—balanced. All the specimens were cut from the substrate of spiral welded pipes made of X80 steel.

The specimens for electrochemical measurements were cut into rectangular pieces of 20 × 20 × 5 mm^3^. For the potentiodynamic polarization test, a hole with a diameter of 5 mm was made in the center of some test pieces upon which a Cu wire was welded to the sample surface. Then, the samples were sealed with epoxy resin. The working surface was sanded with SiC paper (from 400 to 2000 grit), followed by cleaning in acetone and alcohol. 

**Figure 1:**
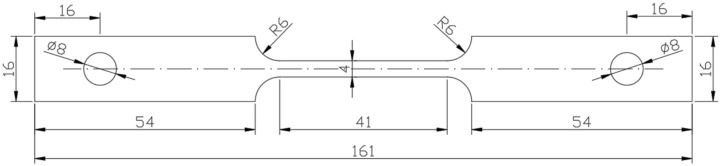
The size (in millimeters) of the SSRT specimen, 2 mm thick.

The dimensions of the SSRT specimens are shown in Figure 1. The thickness of the SSRT specimens was 2 mm. Each specimen was identified with a unique number and sanded in the longitudinal direction of the specimen, using SiC paper up to 2000 grit to ensure the same surface roughness, and then blow-dried after rinsing with distilled water and ethanol.

### 2.2. Corrosion Medium 

The test medium represented the typical soil along the natural gas pipeline in Heilongjiang Province. It was taken from Heihe City, Heilongjiang Province. The composition of the soil was determined according to the methods described in GB/T 6920-1986, GB/T11896-1989, GB/T11905-1989, and other standards. The pH value and the chemical composition of the soil are shown in Table 1. The type of soil was meadow clay. Before testing, the soil was dried at 150 °C for about 12 h, and then screened with 20 mesh sieves to remove the larger particles. The experimental medium was prepared by mixing the dry soil with 40% saturated water. 

### 2.3. Electrochemical Measurements

**Figure 2:**
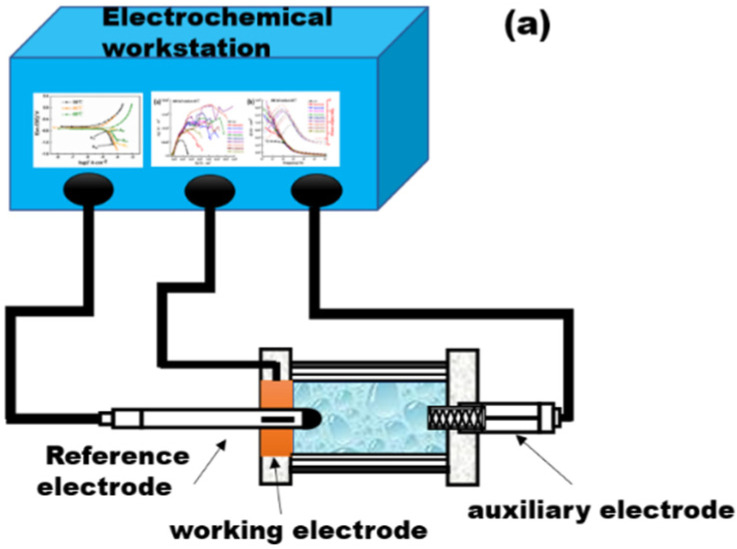

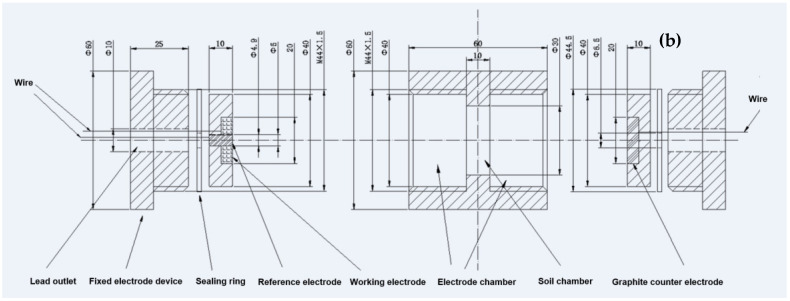
(**a**) Schematic diagram of electrochemical test; (**b**) electrolytic cell designed for electrochemical test.

An electrolytic cell specially designed for the electrochemical test of soil corrosion was used to conduct the electrochemical test. The electrochemical experiment is schematically shown in Figure 2a, and the dimensions of the electrolytic cell apparatus are shown in Figure 2b. In this device, the reference electrode was back-inserted during measurements. By using this device, the potential drop caused by soil resistance was effectively reduced. In addition, the thread and sealing ring components were designed to keep the moisture stable in the device at an elevated temperature for a long time. The electrochemical test was accomplished using a PARSTAT 2273 electrochemical workstation with a conventional three-electrode system. The samples served as the working electrode, the saturated copper sulfate electrode (CSE) was the reference electrode, and the graphite was the auxiliary electrode. The experiment was carried out in accordance with ISO 17475: 2005. The scan interval for the dynamic potential polarization curve experiment was −0.9~+0.9 V relative to the open circuit potential, and the scan rate was 0.5 mV/s.

The EIS measurements were carried out at the open circuit potential (OCP). The signal amplitude applied to this system during the testing process was 10 mV, and the testing frequencies ranged from 10 mHz to 100 kHz. The samples were pre-polarized for 10 min before the EIS test started at each potential. At each potential, three repeat experiments were conducted for all samples to ensure consistency of the data. The EIS data were analyzed using ZSimpWin software. 

### 2.4. SSRT Tests

The SSRT was carried out in a WDML-3 testing machine, which was controlled by a microcomputer. Each specimen was pre-charged in hydrogen gas in the environmental chamber for six hours prior to performing a tensile test. The tensile test started after the testing system was stabilized at the required temperature. The load–deformation curve was automatically collected and recorded by the computer. 

The SSRT was carried out in line with standard ISO 7539-7-2005. The strain rate was set as 10^−6^/s, and a tensile test performed in air medium was used as a reference test. The specimens tested at different cathodic potentials were all polarized for 6 h in advance at the corresponding cathodic potential before testing to ensure the hydrogen atoms generated had enough time to diffuse into the samples.

### 2.5. SEM Surface Morphologies

After SSRT, the fracture surfaces of the specimens were subsequently cleaned using a cleaning agent (IS-129 + HCl), distilled water, and alcohol. Thereafter, the micro morphologies of the fracture surfaces were observed using a scanning electron microscope (SEM, Philips XL 30-FEG, Amsterdam, The Netherlands).

## 3. Results and Discussion

### 3.1. CP Potential of X80 Steel at Different Temperatures

#### 3.1.1. Potentiodynamic Polarization Test

**Figure 3:**
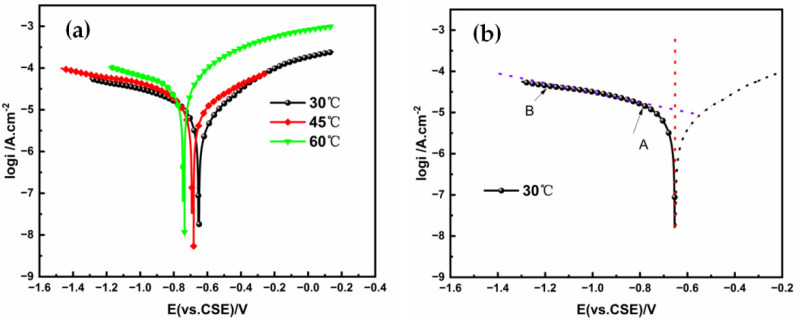
(**a**) Cathodic polarization curves of X80 at 30 °C; (**b**) Tafel polarization curves of X-80 in soil extracts at different temperatures.

Figure 3a shows the X80 cathodic polarization curve at 30 °C. Two characteristic points, A and B, can be found in the curve. Near these two points, the slope of the polarization curve changes. A similar observation can also be found in the potential polarization curves of X80 in soil solution at 30 °C, 45 °C, and 60 °C, in Figure 3b. Point A is the transition point from the oxygen activation control to the oxygen diffusion control. The characteristic Point A was ascribed to the electrochemical reactions on the steel surface [26,27]. At the early stage of cathodic polarization (from the OCP to the Point A stage), the main reaction on the surface of the cathode was the oxygen reduction reaction (ORR), as shown in Equation (1). At this stage, the oxygen reduction was controlled by the rate of charge transfer. With the negative shift in the applied potential in the system, the controlling factor for the cathodic reaction changed from the activated charge transfer of oxygen to the mixed control of the charge transfer and the oxygen diffusion (between A and B). A plateau stage appeared after the second characteristic point (B), where the current density changed slowly with the increasing cathodic potential. At this time, the cathodic reaction rate was controlled by oxygen diffusion. The CP potential was chosen according to the plateau area (from A to B), where the current can be used effectively.

O_2_ + 2H_2_O + 4e → 4OH^−^ (1)

However, the current density increased more slowly with the increasing cathodic potential after B, which was attributed to the hydrogen evolution reaction, as shown in Equation (2), with a continuous negative shift of applied potential [28]. If the CP potential was chosen at this range, the samples might have been damaged due to hydrogen embrittlement and overprotection. Therefore, the appropriate CP potential can be chosen in the range between Points A and B.

2H^+^ + 2e → H_2_ (2)

In Figure 3, the polarization curves of X80 pipeline steel in the soil environment were detected at 30 °C, 45 °C, and 60 °C. It can be seen from the figure that the corrosion potentials of the samples decreased with the increasing temperature from −0.63 V at 30 °C to −0.69 V at 45 °C, and then to −0.75 V at 60 °C. However, the cathodic current density for the samples increased with the increasing temperature. In addition, the hydrogen evolution potential (potential at B) also positively shifted slightly with the increasing temperature from −1.21 V at 30 °C to −1.20 V at 45 °C, and then to −1.19 V at 60 °C. This increase was related to the reduction in the oxygen content and the enhanced hydrogen content in the soil with the increasing temperature. When the soil temperature increased, the transportation rate of hydrogen ions accelerated, and thus the hydrogen evolution rate increased [29,30]. Accordingly, the CP potential for X80 can be chosen in the range of −800 mV to −1200 mV (vs. CSE) temporally. 

#### 3.1.2. EIS

**Figure 4:**
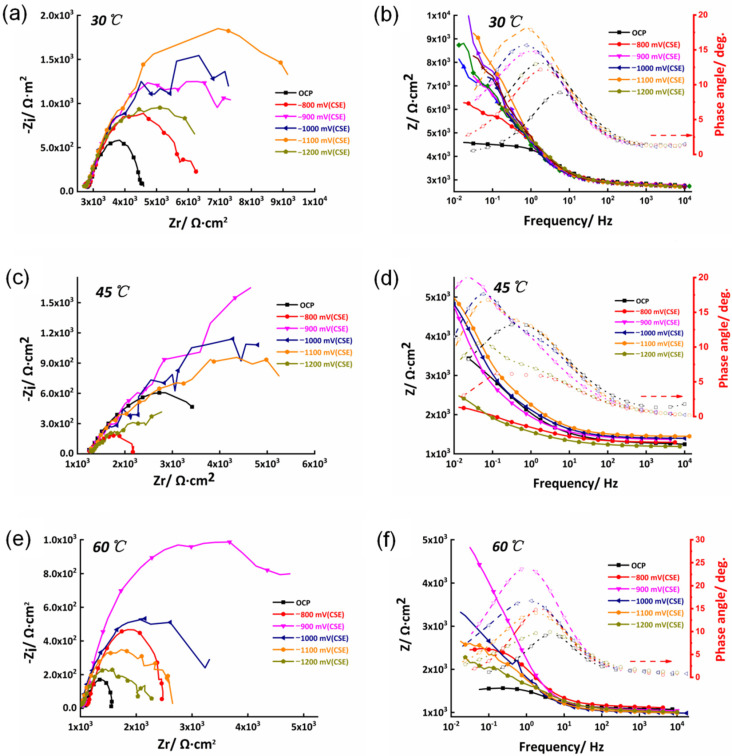
The EIS of the X80 steel under different cathodic polarization potentials and characteristic frequencies at (**a**,**b**) 30 °C, (**c**,**d**) 45 °C, and (**e**,**f**) 60 °C.

The EIS test was conducted under open circuit potential, and different cathodic polarized potentials that were within the potential range were investigated. Figure 4 presents the EIS curves of the X80 steel measured at different CP potentials at 30 °C, 45 °C, and 60 °C, respectively. It can be seen in Figure 4a that, when the applied CP potential was higher, the anode reaction was suppressed, and the cathodic reaction was mainly controlled by oxygen diffusion. When the cathodic polarization potential decreased to a lower value, the peak potential reached its peak value (−900 mV), where the impedance of this system reached the summit. As the CP potential further decreased, a hydrogen evolution reaction occurred. The peak potential became the actual hydrogen evolution potential, as proven in previous works [31,32]. With the increase in the cathodic over-potential, the characteristic frequency corresponding to the time constant shifted towards a lower frequency (Figure 4b), which is consistent with the change law of the electron transfer resistance. When the temperature of this system increased from 30 °C to 45 °C (Figure 4c,d) and 60 °C (Figure 4e,f), the trend was the same as that at 30 °C, including the peak potentials. 

**Figure 5:**
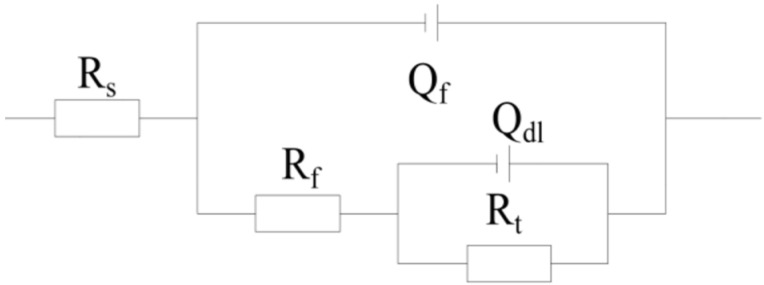
The equivalent circuit fitted by the data from the EIS measurement.

The EIS data are fitted with the equivalent circuit in Figure 5, where *R*_s_ is the solution resistance, and *Q*_f_ and *R*_f_ are the constant phase angle elements and indicate resistance at the interface between the corrosion product and the X80 matrix. The corrosion product originated from the long-time immersion of the samples into the corrosion medium. *Q*_dl_ refers to the constant phase angle element of the double layer at the interface between the corrosion product and the corrosion medium. *R*_t_ represents the charge transfer resistance. 

**Figure 6:**
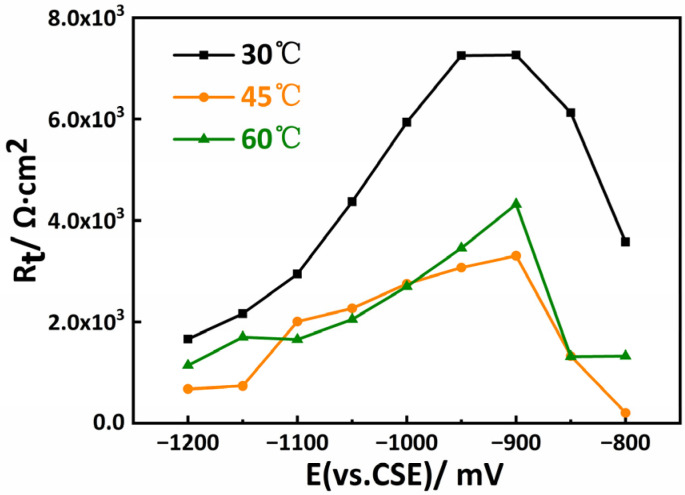
The charge transfer resistance (*R*_t_) versus potential plots for the X80 steel in the soil medium at 30 °C, 45 °C, and 60 °C.

Figure 6 shows the charge transfer resistance (*R*_t_) versus the potential plots extracted from the EIS curves. It can be seen from the figure that the *R*_t_ for the sample measured at 30 °C was much higher than those obtained at higher temperatures. However, the peak potential corresponding to the highest *R*_t_ was −900 mV, which is the same as that of the samples measured at different temperatures.

It can also be observed in Figure 6 that the *R*_t_ value increased as the CP potential increased from −800 mV to −900 mV and then decreased with an increasingly negative CP potential at different temperatures, resulting in peak potentials corresponding to a specific CP potential. As discussed above, the actual hydrogen evolution potentials for the X80 steel in the soil medium at 30 °C, 45 °C, and 60 °C were approximately the same values. As the potential further decreased to −1100 mV, the hydrogen evolution reaction became the main cathodic reaction. A large amount of hydrogen was released while the *R*_t_ remained almost constant and stable during this period. Therefore, according to the potentiodynamic polarization and EIS results, the CP potential was between −900 mV and −1100 mV. 

### 3.2. Hydrogen Embrittlement Sensitivity of X80 Steel

#### 3.2.1. SSRT

**Figure 7:**
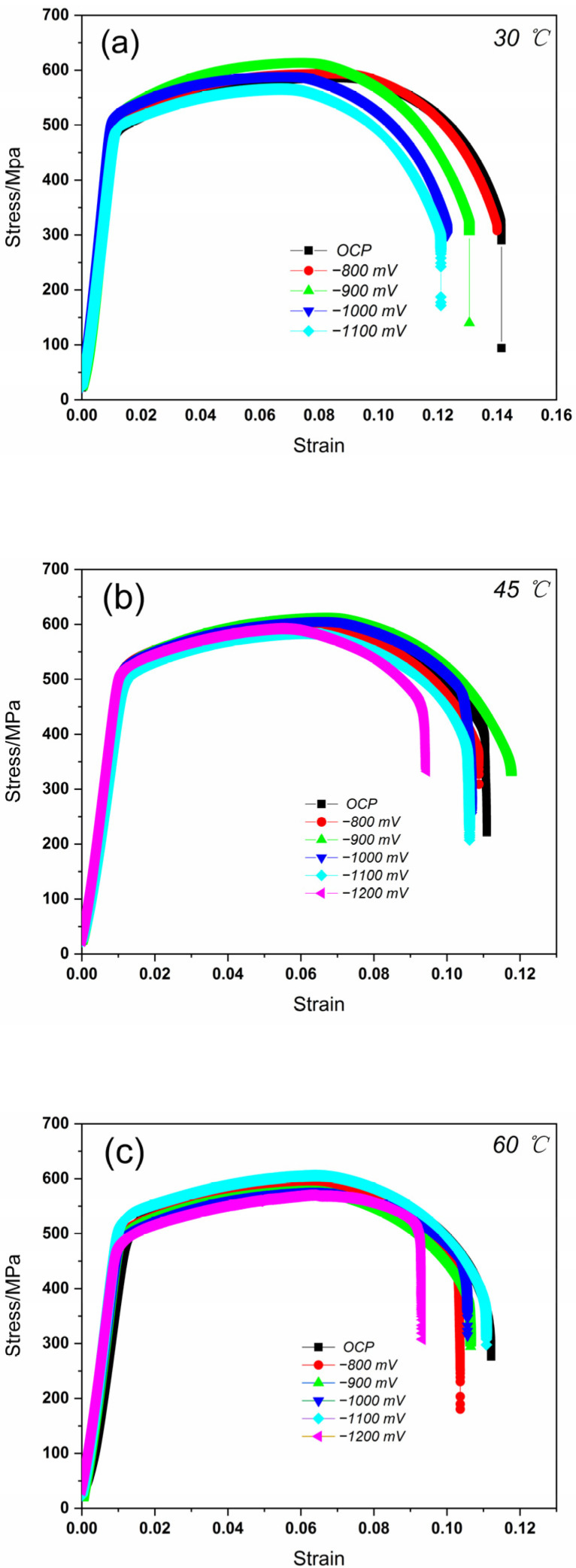
The SSRT curves for X80 steel at different CP potentials in soil medium at (**a**) 30 °C, (**b**) 45 °C, and (**c**) 60 °C.

The SSRT curves for the X80 steel under different CP potentials at 30 °C, 45 °C, and 60 °C are presented in Figure 7. During the elastic strain stage, there were no apparent differences among the different cathodic potentials. When the temperature of the soil medium was 30 °C, the tensile strength of the X80 steel initially decreased with the increasingly negative potential. Based on the EIS results, the peak potential where the X80 steel possessed the best mechanical property corresponded to −900 mV. Once the soil medium temperature increased to 45 °C, the mechanical property of the X80 steel again initially increased and then decreased with the increasingly negative CP potential. The peak potential was about −900 mV at this temperature. Once the applied potential became more negative after passing the peak value, the mechanical properties of this material decreased rapidly. At 60 °C, however, there was little change in the stress–strain curves before −900 mV. The UTS and elongation decreased when the CP potential became more negative. It appears that the hydrogen evolution became significant after −900 mV.

This phenomenon can be explained by the correlation between H atoms and the X80 material [33]. The H atoms can improve the strength of X80 when entered into the matrix because they can gather and produce a Cottrell atmosphere, which can effectively pin the dislocations and improve the mechanical strength of the X80 steel [18]. When the applied potential is relatively positive, a small amount of hydrogen is produced, and the strengthening function is enhanced. With the negative shift of the applied cathodic potential, the hydrogen evolution reaction is enhanced. The break extension decreases with the negative shift in the potential.

**Figure 8:**
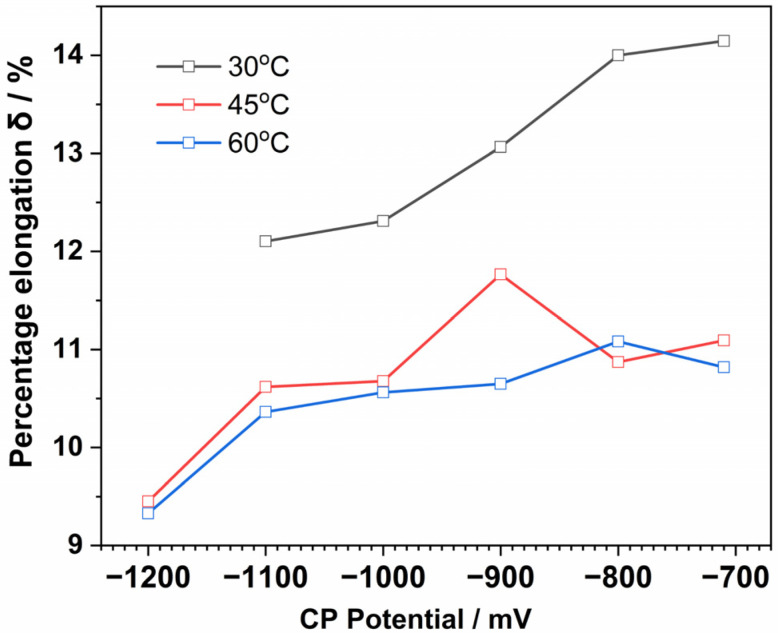
Elongation of the X80 steel under different CP potentials in the soil medium.

Table 2, Table 3 and Table 4 summarize the mechanical property parameters at 30 °C, 45 °C, and 60 °C obtained from the SSRT test results. *σ*_s_ is yield strength and *σ*_b_ is tensile strength. Figure 8 shows the elongation percentage (δ/%) of the samples in the soil environment at different applied CP potentials. As expected, the applied CP potentials significantly influenced the mechanical properties of the X80 steel. The elongation (δ/%) decreased with an increasing applied CP potential, especially when the CP potential reached −1200 mV, revealing the hydrogen embrittlement susceptibility of the steel at a high CP potential. The fracture energy loss (*I*_E_) of the X80 steel increased with an increasingly negative CP potential. Although the increasing environment temperature significantly reduced the elongation of the steel, the same trend is shown for the mechanical properties at all the temperatures.

#### 3.2.2. Fracture Morphology

**Figure 9:**
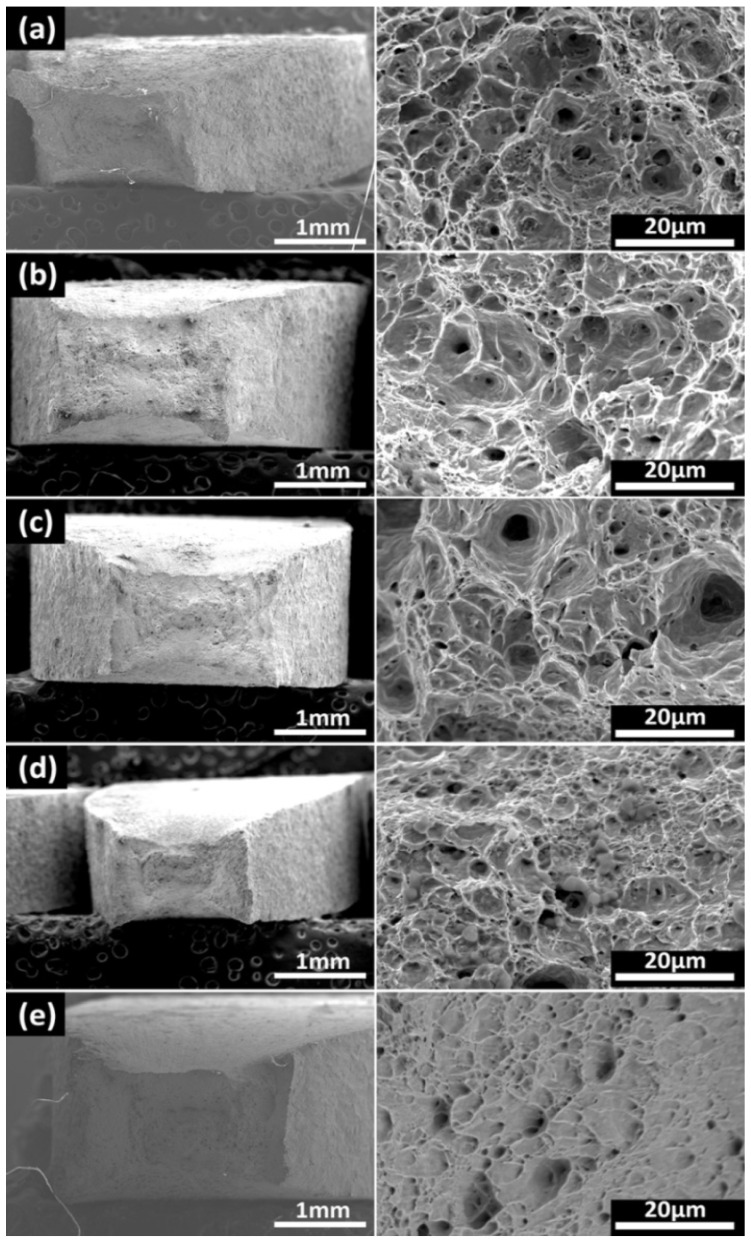
Fracture morphologies for the X80 steel under different CP potentials at 30 °C: (**a**) OCP, (**b**) −800 mV, (**c**) −900 mV, (**d**) −1000 mV, and (**e**) −1100 mV.

**Figure 10:**
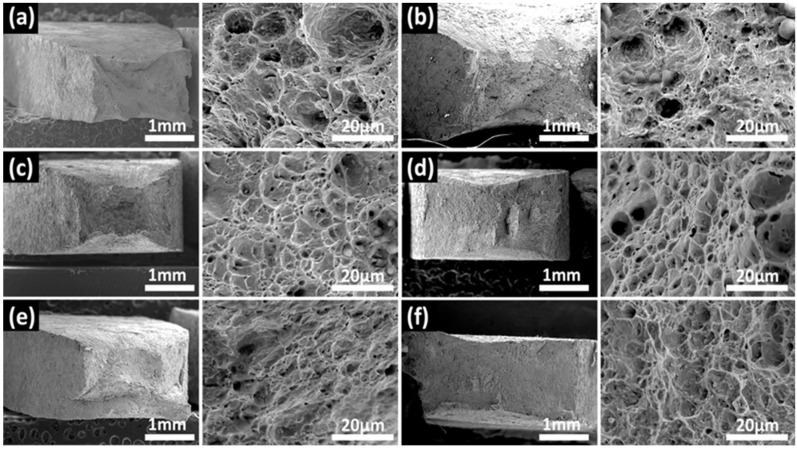
Fracture morphologies for X80 under different CP potentials ranging from OCP to −1200 mV at 45 °C: (**a**) OCP, (**b**) −800 mV, (**c**) −900 mV, (**d**) −1000 mV, (**e**) −1100 mV, (**f**) −1200 mV.

**Figure 11:**
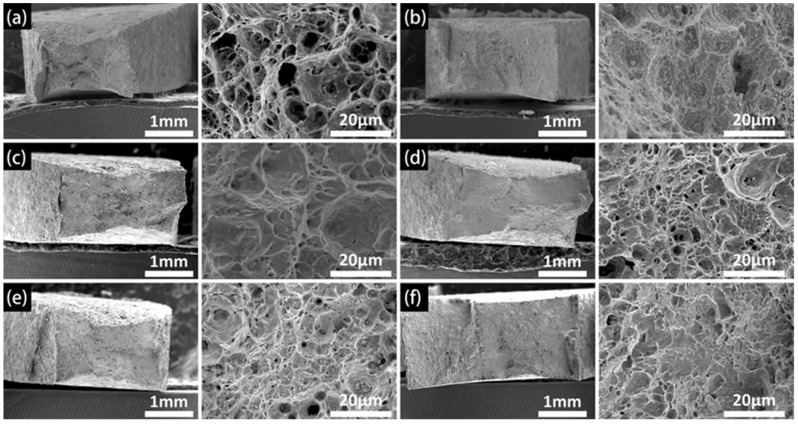
Fracture morphologies for X80 under different CP potentials ranging from OCP to −1200 mV at 60 °C: (**a**) OCP, (**b**) −800 mV, (**c**) −900 mV, (**d**) −1000 mV, (**e**) −1100 mV, (**f**) −1200 mV.

Figure 9, Figure 10 and Figure 11 show the fracture morphologies of the X80 steel after the SSRT tests. The typical ductile fracture characteristics of dimples can be observed on the fracture surfaces. However, this characteristic became implicit when the applied potential became more negative. When applied at the open circuit potential, dimples with different sizes were distributed on the fracture surfaces, as shown in Figure 9a. An obvious necking phenomenon can be seen in this image. It exhibits a typical micro-pore aggregation ductile fracture. After the protection potential was applied to this system, the fracture morphology did not obviously change. The number and the depths of dimples in the fracture surface gradually decreased after the protection potential became more negative than the peak potential, indicating reduced plasticity of the X80 steel. This was more evident at higher temperatures (45 °C and 60 °C), where dimple sizes were significantly reduced at a high negative applied cathodic potential, with the fracture surfaces exhibiting quasi-cleavage fracture morphologies. Furthermore, the necking was also significantly reduced. The limit potential for the obvious transition from the plastic fracture to the quasi-cleavage fracture can be estimated from the images in Figure 8, Figure 9, Figure 10 and Figure 11, which are −1100 mV, −1100 mV, and −1200 mV for the X80 steel at 30 °C, 45 °C, and 60 °C, respectively. The quasi-cleavage fracture was mainly caused by the hydrogen embrittlement mechanism due to the penetration of H atoms into the bulk of the steel, as a previous study suggested [34].

## 4. Conclusions

In this work, the CP criteria and mechanical properties were studied for the X80 steel gas pipeline in soil at different temperatures by measuring the potentiodynamic polarization curves, performing electrochemical impedance spectroscopy, and conducting slow strain tensile rate tests. The results can be summarized as follows:

(1) The appropriate cathodic protection potential for X80 in Heilongjiang soil at the temperatures of concern was established to be between −900 mV (vs. CSE) and −1100 mV (vs. CSE).
(2) The mechanical properties of X80 decreased with an increasing negative cathodic potential due to the absorption of the generated hydrogen into the steel.
(3) The elongation of the SSRT test specimens decreased when the applied CP potential became more negative due to the increasing hydrogen embrittlement susceptibility of the steel at a high CP potential.

## Figures and Tables

**Table 1:** Chemical composition of the dry soil taken from Heihe.

Soil Type	pH	Cl^−^	SO_4_^2−^	HCO_3_^−^	Ca^2+^	Mg^2+^	K^+^	Na^+^
Meadow	6.01	0.044	0.24	0.0725	1.7	1.6	19	1.3

**Table 2:** The tensile properties of X80 steel under different CP potentials in soil medium at 30 °C.

E_(CSE)_/mV	*σ*_b_/Mpa	*σ*_s_/Mpa	Fracture Energy E/(J·cm^−2^)	δ/%	ψ/%	Fracture Energy Loss (*I*_E_)/%
OCP	585.913	494.838	73.2233	14.146	65.00	1.0070
−800	593.713	511.225	73.5343	14.001	67.24	0.00586
−900	611.113	523.113	69.5261	13.065	74.11	6.0053
−1000	590.538	519.200	63.5686	12.311	68.32	14.0594
−1100	565.813	500.550	59.4923	12.103	67.36	19.5703

**Table 3:** The tensile properties of X80 under different CP potentials in soil medium at 45 °C.

E_(CSE)_/mV	*σ*_b_/Mpa	*σ*_s_/Mpa	Fracture Energy E/(J·cm^−2^)	δ/%	ψ/%	Fracture Energy Loss (*I*_E_)/%
OCP	587.850	512.875	56.4625	11.093	57.265	23.0566
−800	602.463	529.488	57.3156	10.873	60.625	20.2727
−900	609.888	526.938	62.5581	11.766	65.35	27.0676
−1000	607.688	528.188	57.2155	10.678	34.75	30.0478
−1100	584.288	509.050	54.0241	10.619	57.565	26.8721
−1200	592.638	526.825	49.2806	9.451	34.9	35.3617

**Table 4:** The tensile properties of X80 under different CP potentials in soil medium at 60 °C.

E_(CSE)_/mV	*σ*_b_/Mpa	*σ*_s_/Mpa	Fracture Energy E/(J·cm^−2^)	δ/%	ψ/%	Fracture Energy Loss (*I*_E_)/%
OCP	592.688	512.588	56.9136	10.819	56.800	23.6664
−800	606.050	531.000	58.9728	11.082	50.200	22.5131
−900	576.075	503.650	53.9467	10.650	45.4	15.4256
−1000	579.600	495.838	51.7423	10.563	41.4	22.6625
−1100	587.138	505.913	54.0913	10.364	25.525	26.9807
−1200	570.438	494.750	47.8117	9.329	31.112	33.3880
